# Radial artery access is associated with lower mortality in patients undergoing primary PCI: a report from the SWEDEHEART registry

**DOI:** 10.1177/2048872620908032

**Published:** 2020-10-07

**Authors:** Christian Dworeck, Björn Redfors, Sebastian Völz, Inger Haraldsson, Oskar Angerås, Truls Råmunddal, Dan Ioanes, Anna Myredal, Jacob Odenstedt, Geir Hirlekar, Sasha Koul, Ole Fröbert, Rickard Linder, Dimitrios Venetsanos, Robin Hofmann, Anders Ulvenstam, Petur Petursson, Giovanna Sarno, Stefan James, David Erlinge, Elmir Omerovic

**Affiliations:** 1 Department of Cardiology, Sahlgrenska University Hospital, Sweden; 2 Department of Cardiology, Clinical Sciences, Lund University, Sweden; 3 Department of Cardiology, Örebro University, Sweden; 4 Department of Cardiology, Karolinska University Hospital, Sweden; 5 Department of Clinical Science and Education, Karolinska Institutet, Sweden; 6 Department of Cardiology, Östersund Hospital, Sweden; 7 Department of Medical Sciences and Uppsala Clinical Research Center, Uppsala University, Sweden

**Keywords:** ST-elevation myocardial infarction, primary PCI, arterial access site

## Abstract

**Objectives:**

The purpose of this observational study was to evaluate the effects of radial artery access versus femoral artery access on the risk of 30-day mortality, inhospital bleeding and cardiogenic shock in patients with ST-elevation myocardial infarction undergoing primary percutaneous coronary intervention.

**Methods:**

We used data from the SWEDEHEART registry and included all patients who were treated with primary percutaneous coronary intervention in Sweden between 2005 and 2016. We compared patients who had percutaneous coronary intervention by radial access versus femoral access with regard to the primary endpoint of all-cause death within 30 days, using a multilevel propensity score adjusted logistic regression which included hospital as a random effect.

**Results:**

During the study period, 44,804 patients underwent primary percutaneous coronary intervention of whom 24,299 (54.2%) had radial **access** and 20,505 (45.8%) femoral access. There were 2487 (5.5%) deaths within 30 days, of which 920 (3.8%) occurred in the radial access and 1567 (7.6%) in the femoral access group. After propensity score adjustment, radial access was associated with a lower risk of death (adjusted odds ratio (OR) 0.70, 95% confidence interval (CI) 0.55–0.88, *P* = 0.025). We found no interaction between access site and age, gender and cardiogenic shock regarding 30-day mortality. Radial access was also associated with a lower adjusted risk of bleeding (adjusted OR 0.45, 95% CI 0.25–0.79, *P* = 0.006) and cardiogenic shock (adjusted OR 0.41, 95% CI 0.24–0.73, *P* = 0.002).

**Conclusions:**

In patients with ST-elevation myocardial infarction, primary percutaneous coronary intervention by radial access rather than femoral access was associated with an adjusted lower risk of death, bleeding and cardiogenic shock. Our findings are consistent with, and add external validity to, recent randomised trials.

## Introduction

Primary percutaneous coronary intervention (PCI) by radial artery access (RA) rather than femoral artery access (FA) has been shown in recent multicentre randomised trials to reduce the risk of death and several other adverse clinical events among patients with ST-elevation myocardial infarction (STEMI).^1–3^ However, the patients who were included in these trials represent a selected population, and the external validity of the findings in these trials for an unselected cohort of patients who undergo coronary angiography due to STEMI has been questioned.^4^ Whereas the current European Society of Cardiology (ESC) guideline on the management of patients with STEMI has a strong recommendation for RA,^5^ the American STEMI guideline does not.^6^ This difference between the European and American guidelines is reflected in current clinical practice by the fact that RA has become the default strategy in many European countries,^7–11^ whereas FA remains the default strategy for many American operators. However, there are also large variations in the preferred access site across different operators and hospitals within European countries as well as within the USA.^12,13^

Our aim was to assess whether the benefits with RA in patients with STEMI undergoing PCI observed in recent randomised clinical trials are reproducible in a nationwide Swedish population of unselected STEMI patients.

## Methods

### Databases and patient selection

The Swedish Coronary Angiography and Angioplasty Registry (SCAAR) gathers data on all consecutive patients from all hospitals performing coronary angiography and PCI in Sweden. It was established in 1999 and is now part of the national SWEDEHEART (Swedish Web–System for Enhancement and Development of Evidence-Based Care in Heart Disease Evaluated According to Recommended Therapies) registry. The registry is sponsored solely by the Swedish health authorities and receives no commercial funding. The registry’s technology was developed and is administered by the Uppsala Clinical Research Centre. Since 2001, SCAAR has used a web-based case report platform with automatic data surveillance. In total, 30 hospitals in Sweden, including nine university hospitals, have cardiac catheterisation facilities. In SCAAR, a coronary angiographic procedure is described by about 50 variables and a PCI procedure by about 200 variables. After reviewing the clinical information, the PCI physician immediately enters clinical characteristics and procedural details into the registry. SCAAR obtains data on patients’ vital status continuously from the national death registry which, due to the use of mandatory personal identification numbers, has a very high degree of completeness, but it is not reviewed or adjudicated to establish the cause of death. The study population consisted of all patients who were treated by primary PCI for STEMI in Sweden between January 2005 and December 2016. Patients who underwent coronary angiography for presumed STEMI without following PCI (coronary artery bypass grafting (CABG) or no revascularisation) are not included. Patients were stratified according to whether they had RA or FA. Analysis was intention-to-treat (conversion from RA to FA was analysed as RA and vice versa).

### Statistics

Adjustments for differences in baseline characteristics were made with the propensity score. The following variables were included in the calculation of the propensity score: age, gender, smoking habits, hypertension, diabetes, hyperlipidaemia, severity of coronary artery disease, previous infarction, previous PCI, previous CABG, anticoagulation therapy with glycoprotein IIb/IIIa receptor antagonists (GP IIb/IIIa), bivalirudin, P2Y12 antagonist, unfractionated heparin (UH)/low-molecular weight heparins (LMWHs), drug-eluting stents (DESs), completeness of revascularisation, number of stents, type of lesion, reperfusion time, pretreatment with P2Y12 antagonist, regular versus off-hours, calendar year, hospital and pharmacological treatment after discharge.

The estimated propensity score was then used for Kernel-based matching^14^ (based on Epanechnikov function and bandwidth of 0.06) in multilevel logistic regression which was the primary statistical model. The two groups were compared using multilevel logistic regression, with hospital as a random effect, to account for the hierarchical structure of the database. We imputed missing data with multiple imputation and the chain-equation method,^15,16^ with five datasets. We included an indicator of missingness, an event indicator and calendar year as regular variables,^17^ and imputed continuous variables by ordinary least-squares multiple regression, binary variables by logistic regression, and categorical variables by multinomial logistic regression. The imputation procedure and subsequent analyses were done according to Rubin’s protocol^18^ under the assumption that missing data are missing at random.

Our primary hypothesis was that RA in primary PCI reduces all-cause mortality. The primary endpoint was death at 30 days post PCI. Our secondary hypothesis was that RA in primary PCI reduces inhospital bleeding, stroke and cardiogenic shock (CS). Secondary endpoints were inhospital stroke, inhospital bleeding (defined as bleeding mandating transfusion or operation, cardiac tamponade, fall of haemoglobin more than 20 g/L, haematoma larger than 5 cm, compression time >6 hours, premature cessation of antithrombotic treatment due to bleeding, bleeding mandating other treatment than only local compression, prolongation of hospitalisation, or inctracranial bleeding) or CS (defined as Killip class IV). Patients who had CS at the time of admission to the coronary care unit were excluded from the statistical model in which CS was the outcome variable.

## Results

### Patient characteristics and treatments

**Figure 1.**
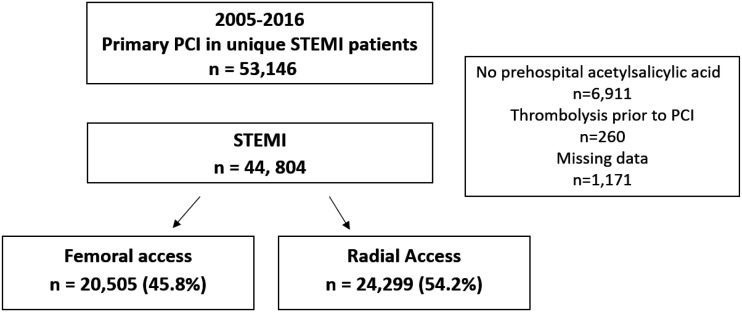
Flow chart for patient selection in Swedish Coronary Angiography and Angioplasty Registry (SCAAR).

**Figure 2.**
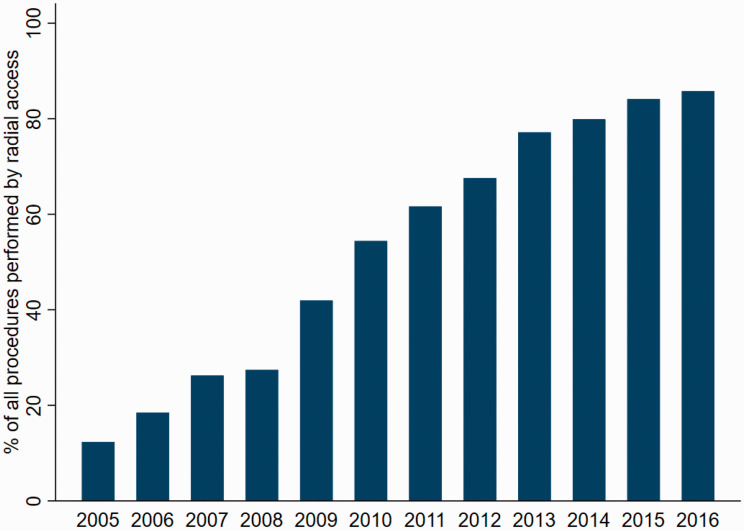
The number of primary percutaneous coronary intervention (PCI) procedures performed by radial access per calendar year in Sweden between 2005 and 2016.

We identified 53,146 patients who underwent primary PCI during the study period (Figure 1). We excluded patients who did not receive acetylsalicylic acid before PCI (*N* = 6911), patients who were treated with thrombolysis (*N* = 260) and patients with missing data that were not imputed (*N* = 1171). The remaining 44,804 patients (28.6% women) were included in the study, of whom 24,299 (26.8% women) had RA. These patients were reported from 30 different hospitals, and the range of reported patients per hospital was 102 to 6471. Between 2005 and 2016, the number of RA increased by 35% per year from 12.3% in 2005 to 85.8% in 2016 (*P* < 0.001, Figure 2). The characteristics of the patients are presented in Table 1 and procedure related details in Table 2. Patients with a RA access were on average younger, more likely to have heart failure and less likely to have hyperlipidaemia, myocardial infarction, stroke, prior PCI or prior CABG. During PCI, RA patients were more often treated with ticagrelor, prasugrel and bivalirudin but less often with a GP2b/3a receptor antagonist and unfractionated heparin. RA patients were also less likely to have complex coronary artery disease. They were more likely to have complete revascularisation during the index PCI with DESs. Stents were more often placed without prior balloon dilation of the lesion in RA patients. The median time from symptom debut to the first medical contact and from the first medical contact to the start of PCI in the total population was 113 minutes (interquartile range (IQR) 50–293) and 74 minutes (IQR 48–125), respectively, and these times did not differ significantly between the two groups (Table 2). After adjustment for propensity score, the two groups were well balanced.

**Table 1.** Patient characteristics.

	Femoral (*N* = 20,505)	Missing	Radial (*N* = 24,299)		Missing		Standardised difference	Standardised difference after adjustment
Age (years)								
Mean age, years	68 ± 12	0		67 ± 12		0	0.036	0.097
Age >75, *n* (%)	5895 (28.6)	0		6232 (25.7)		0	0.065	0.087
Male sex, *n* (%)	14,202 (69.3)	0		17,780 (73.2)		0	0.084	0.107
Diabetes, *n* (%)	3185 (15.5)	0		3608 (14.6)		0	0.014	0.028
Hypertension, *n* (%)	9353 (45.6)	0		11,153 (45.9)		0	0.006	0.074
Smoking, *n*/total *n* (%)		2350 (11.4)				1631 (6.7)		
Never smoker	8249 (40.1)			9652 (39.7)			0.009	0.002
Previous smoker	5978 (29.5)			7362 (30.3)			0.025	0.017
Current smoker	6274 (30.6)			7281 (30.0)			0.015	0.014
Hyperlipidaemia, *n* (%)	5580 (27.2)	0		5859 (24.1)		0	0.071	0.061
Previous stroke, *n* (%)	1386 (6.8)	1992 (9.7)		1222 (5.1)		3496 (14.4)	0.074	0.066
History of heart failure, *n* (%)	998 (4.9)	1530 (7.5)		1576 (6.5)		3939 (16.2)	0.07	0.071
Previous myocardial infarction, *n* (%)	4170 (20.3)	0		3545 (14.6)		0	0.152	0.079
Previous PCI — n (%)	2989 (14.6)	0		2887 (11.9)		0	0.08	0.008
Previous CABG, *n* (%)	1200 (5.6)	0		396 (1.6)		0	0.224	0.028
Symptom to first contact, min; median (IQR)	110 (50–268)	0		115 (50–317)		0	0.016	0.001
First contact to start of PCI, min; median (IQR)	73 (45–125)	0		75 (50–126)		0	0.019	0.015
Prehospital heparin	4586 (22.6)	0		12,925 (53.2)		0	0.675	0.101
Days in hospital, mean (±SD)	5.1 ± 14.7	0		4.6 ± 5.5		0	0.120	0.131
Medications used at time of presentation, *n* (%)								
Beta-blockers	6549 (31.9)	623 (1.6)		7463 (30.7)	91 (1.3)		0.024	0.074
ACE inhibitor	3374 (16.6)	607 (1.6)		4570 (18.8)	85 (1.2)		0.063	0.027
ARB receptor antagonist	2220 (10.8)	602 (1.6)		3422 (14.8)	88 (1.3)		0.097	0.015
Acetylsalicylic acid	6604 (32.2)	414 (1.1)		7528 (30.9)	73 (1.1)		0.023	0.073
P2Y12 receptor antagonist	1234 (6.1)	3971 (10.5)		1073 (4.4)	1036 (14.9)		0.071	0.045
Statin	5067 (24.7)	423 (1.1)		6826 (28.1)	75 (1.1)		0.076	0.031
OAC or NOAC	359 (1.6)	56 (0.2)		471 (1.9)	27 (0.4)		0.017	0.018

PCI: percutaneous coronary intervention; CABG: coronary artery bypass grafting; CPR: cardiopulmonary resurrection; ACE: angiotensin-converting enzyme inhibitor; ARB: angiotensin receptor blocker; OAC: oral anticoagulant; NOAC: novel oral anticoagulant.

**Table 2.** Angiography and PCI.

	Femoral (*N* = 20,505)	Missing	Radial (*N* = 24,299)	Missing	Standardised difference	Standardised difference after adjustment
Procedure performed off-hours, *n* (%)	12,722 (64.6)	807 (3.9)	15,986 (65.8)	392 (1.6)	0.046	0.006
Infarct-related artery, *n*/total *n* (%)		359 (1.8)		140 (2.0)		
RCA	8002 (39.7)		8889 (37.2)		0.050	0.126
LAD	8695 (43.2)		10,873 (45.6)		0.047	0.119
LCx	3183 (15.8)		3876 (16.2)		0.001	0.035
LM	262 (1.3)		229 (0.96)		0.022	0.105
Arteries with stenosis, *n*/total *n* (%)		130 (0.6)		31 (0.1)		
0	158 (0.8)		183 (0.6)		0.006	0.001
1	9186 (44.8)		12,546 (51.6)		0.127	0.041
2 or 3 no LM	9815 (47.8)		10,615 (43.7)		0.087	0.018
LM and 1, 2 or 3	1212 (5.9)		920 (3.8)		0.095	0.054
Complete revascularization, *n*/total *n* (%)	10,381 (50.6)	188 (0.9)	14,779 (60.8)	256 (1.3)	0.204	0.067
Type of lesion		126 (0.6)		38 (0.2)		
A	1510 (7.4)		1694 (7.0)		0.018	0.020
B1	5617 (27.6)		7007 (28.9)		0.027	0.030
B2	6734 (33.3)		8946 (36.9)		0.076	0.011
C	4666 (23.1)		4177 (17.3)		0.144	0.080
B1 bifurcation	384 (1.9)		663 (2.7)		0.053	0.033
B2 bifurcation	754 (3.7)		1138 (4.7)		0.047	0.028
C bifurcation	569 (2.8)		593 (2.6)		0.022	0.020
Type of stenosis		8 (0.02)		3 (0.04)		
De novo	19,241 (93.9)		23,334 (96.0)		0.097	0.065
In-stent	1078 (5.3)		844 (3.5)		0.042	0.012
Other	172 (0.8)		116 (0.5)		0.087	0.065
PCI with stent, *n*/total *n* (%)		2 (0.001)		0 (0.01)		
Drug-eluting stent	5921 (28.9)		15,120 (62.2)		0.648	0.017
Bare metal stent	12,722 (62.1)		7690 (31.7)		0.707	0.045
No stent	1856 (9.0)		1485 (6.1)		0.095	0.114
P2Y12 receptor antagonist*		0		524 (7.5)		
Clopidogrel	16,255 (80.8)		9875 (40.1)		0.892	0.038
Ticagrelor	3148 (15.6)		12,642 (52.4)		0.838	0.061
Prasugrel	723 (3.6)		1629 (6.8)		0.141	0.045
Thrombus aspiration, *n* (%)	4603 (22.5)	75 (0.36)	5354 (22.1)	37 (0.15)	0.064	0.031
Direct stenting, *n* (%)	2860 (16.1)	0	3994 (18.1)	0	0.124	0.096
Bivalirudin, *n* (%)	6869 (34.9)	817 (3.94)	12,818 (53.0)	129 (0.53)	0.504	0.061
GP2b/3a receptor inhibitor, *n* (%)	9710 (47.5)	0	5599 (23.1)	0	0.365	0.007
Unfractionated heparin, *n* (%)	11,890 (58.0)	11 (0.04)	15,704 (64.6)	6 (0.02)	0.131	0.017

IRA: infarct-related artery; PCI: percutaneous coronary intervention; RCA: right coronary artery; LAD: left anterior descending artery; LCx: left circumflex artery; LM: left main.

### Clinical outcomes

**Figure 3.**
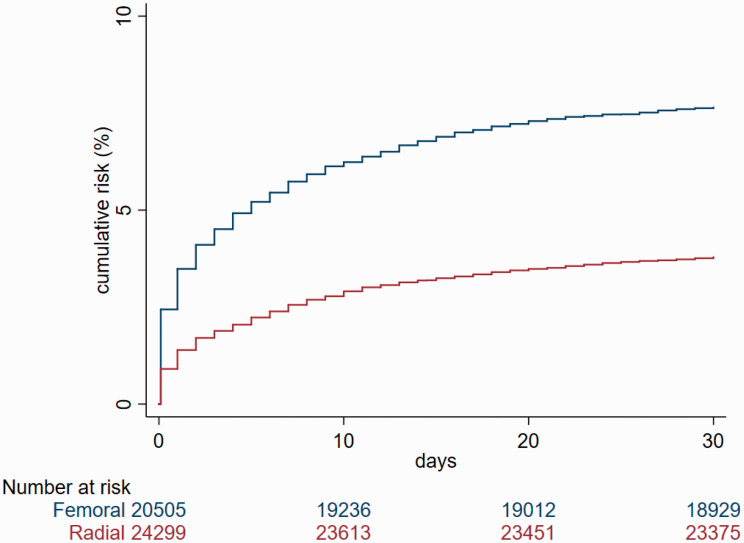
Cumulative incidence of the primary endpoint in relation to arterial access site.

There were 2487 (5.5%) deaths within 30 days, of which 920 (3.8%) occurred in the RA and 1567 (7.6%) in the FA groups (Figure 3). The unadjusted odds ratio (OR) associated with RA versus FA was 0.47 (95% confidence interval (CI) 0.44–0.51) for death within 30 days, 0.56 (95% CI 0.45–0.70) for inhospital bleeding, 0.29 (95% CI 0.23–0.37) for CS and 0.65 (95% CI 0.48–0.87) for stroke. After propensity score adjustment (Table 3), RA was associated with a lower risk of death (adjusted OR 0.70, 95% CI 0.55–0.88, *P* = 0.025), a lower risk of inhospital bleeding (adjusted OR 0.45, 95% CI 0.25–0.79, *P* = 0.006) and a lower risk of CS after PCI (adjusted OR 0.41, 95% CI 0.24–0.73, *P* = 0.002). After adjustment, access site did not modify the risk of stroke (adjusted OR 0.85, 95% CI 0.26–2.84, *P* = 0.797). We found no interaction between access site and age, gender and CS regarding 30-day mortality and bleeding. Exclusion of patients with CS did not substantially change the estimated risk of death at 30 days (adjusted OR 0.58, 95% CI 0.49–0.76, *P* < 0.001).

**Table 3.** Primary analysis.

	Femoral (*N* = 20,505)	Radial (*N* = 24,299)	Adjusted OR	95% CI	*P* value	Missing *n* (%)
Primary endpoint						
Death at 30 days, *n* (%)	1567 (7.6)	920 (3.8)	0.70	0.55–0.88	0.025	0
Secondary endpoints						
Definite stent thrombosis at 30 days, *n* (%)	157 (0.8)	110 (0.5)	1.19	0.50–2.83	0.718	0
Cardiogenic shock, *n* (%)	1044 (5.1)	366 (1.5)	0.41	0.24–0.73	0.002	0
Inhospital bleeding	718 (3.6)	486 (2.1)	0.45	0.25–0.79	0.006	1278 (2.9)
Inhospital neurological complications	67 (0.3)	51 (0.2)	0.83	0.30–2.28	0.721	1002 (2.2)

OR: odds ratio; CI: confidence interval.

## Discussion

The most important finding in our study from the nationwide SWEDEHEART registry is that among 44,804 unselected and consecutive patients with STEMI, coronary angiography and PCI by RA rather than FA access was associated with significantly lower adjusted risks of 30-day mortality, inhospital bleeding and CS. The results of our study are consistent with, and add external validity to, the findings of three recent large randomised trials done on this topic, the RIVAL,^3^ RIFLE-STEACS^1^ and MATRIX^2^ studies. We present one of the largest observational studies on radial versus femoral access in STEMI patients and our findings are congruent with earlier observational studies.^19–24^

We found that RA, as compared to FA, in patients with STEMI is associated with a considerably reduced risk of dying. This finding is consistent with the findings of the three largest randomised trials that compared RA to FA in patients with acute coronary syndromes undergoing invasive management, the MATRIX, RIVAL-STEACS and RIFLE trials.^2^ MATRIX, which was the largest of the three trials, randomly allocated 8404 patients with acute coronary syndromes to either RA or FA. MATRIX reported a relative risk reduction for 30-day mortality with RA versus FA of 0.72, which is similar to the adjusted risk reduction observed in our study. In MATRIX, the risk reduction in 30-day mortality was partly responsible for the risk reduction observed with RA versus FA with regard to the primary composite endpoint all-cause death, myocardial infarction, stroke or the Bleeding Academic Research Consortium (BARC)^25^ level 3 or 5 bleeding. Our results are also consistent with RIFLE-STEACS,^1^ which randomly allocated 1001 patients with STEMI to RA versus FA in four high-volume centres. RIFLE-ACS reported a significant reduction in the risk of the primary composite endpoint 30-day net adverse cardiac event (cardiac death, stroke, myocardial infarction, target lesion revascularisation and non-CABG bleeding), which was partly driven by a significant reduction from 9.2% to 5.2% in the secondary endpoint 30-day cardiac mortality. Whereas the RIVAL study did not show a significant reduction in the composite primary endpoint (30-day death, myocardial infarction, stroke, non-CABG major bleeding) between RA and FA among 7021 patients with acute coronary syndrome, a prespecified subgroup analysis of the 1958 patients with STEMI showed a reduction in the risk of the primary outcome with RA versus FA.^3^

There are several possible explanations for the observed reduction in mortality risk with RA versus FA. The observed reduction in mortality with RA versus FA could be related to a reduced risk of bleeding. It is well known that the risk of significant bleeding complications, particularly those directly related to vascular access, is reduced with RA compared to FA. The inhospital bleeding risk was substantially lower with RA than FA in our study, as well as in each of the randomised multicentre trials.^1,2^ Bleeding complications after PCI, irrespective of whether they are related to the access site,^26–29^ are associated with an increased risk of dying.^27,28,30^ In addition to direct life-threatening complications such as haemorrhagic shock, bleeding increases the risk of both myocardial infarction and ischaemic stroke.^25,28,30–34^ The increased risks of myocardial infarction and stroke for patients who bleed are most likely to be related to cessation of antithrombotic medications, adverse reactions to blood transfusions, activation of the coagulation cascade or by reducing oxygen delivery to already ischaemic tissue. Irrespective of the exact mechanisms through which bleeding after PCI can lead to death, the risk of dying has been shown to be at least as high for patients who have a significant bleed after PCI as for those who have a myocardial infarction after PCI.^27,28,30^ In light of the strong association between bleeding after PCI and mortality, it is not surprising that strategies to reduce bleeding after PCI have been shown to increase survival,^35–37^ with the greatest benefit observed in patients with a relatively high bleeding risk, such as STEMI patients.^27^ Another life-threatening complication that was less likely to occur with RA than FA in our study, and therefore could mediate the reduction in mortality, was CS. The observed RA-associated reduction in the risk of CS is a novel finding because CS was not reported in either MATRIX, RIFLE-ACS or RIVAL, or any of the previously published larger observational studies.^1–3,21–24^ The exact mechanisms linking RA to a reduced risk of CS in STEMI are not immediately evident from our analysis. However, the observed RA-associated reduction in the risk of CS mirrors the RA-associated risk reduction for bleeding, and several studies have reported a strong association between bleeding risk and the risk of CS.^38–42^ In the CRUSADE registry,^41^ patients with major bleeding had a five times higher risk of CS. However, neither CRUSADE nor the other studies were able to establish whether bleeding caused CS,^38–40,42^ whether CS caused bleeding, or to what extent the association between bleeding and CS is explained by other factors. In our study it is more likely that a bleeding event caused CS than vice versa, because only patients who developed CS after PCI were included in the analysis pertaining to the risk of CS.

Irrespective of the underlying mechanisms, the observed association between access site and the risk of CS is an intriguing hypothesis-generating observation which we believe merits further investigation.^43,44^ A third possible mediator of the reduction in mortality risk with RA versus FA is the recently described lower risk of acute kidney injury, which is associated with the increased mortality risk^45^ with RA versus FA. A lower risk of acute kidney injury with RA versus AF has been described for acute coronary syndrome patients^46^ and may be particularly pronounced in STEMI patients.^47,48^ A lower risk of acute kidney injury with RA than FA has been suggested to be related to a more extensive mobilisation of endothelial progenitors after radial versus femoral arterial puncture, due to the smaller vessel diameter of the radial artery.^49–51^ Unfortunately, SWEDHEART does not contain data on the rate of acute kidney injury after PCI.

### Study limitations

This was an observational study and provides only evidence of association, not cause. We cannot exclude residual confounding or selection bias. A proportion of patients had missing data. We do not have data on cause-specific mortality. We have no data on blood pressure or heart rate on admission, that is, factors independently associated with the development of CS^52^ and patients in Killip classes II–III were not excluded.

In conclusion, in patients with STEMI, primary PCI via RA rather than FA was associated with an adjusted lower risk of death, bleeding and CS. Our findings are consistent with, and add external validity to, recent randomised trials, and support the ESC guideline class Ia recommendation for the use of radial access for primary PCI in STEMI.
